# A Flow Cytometry Study of the Binding and Stimulation Potential of Inactivated *Trypanosoma evansi* toward Dromedary Camel Leukocytes

**DOI:** 10.3390/pathogens13010021

**Published:** 2023-12-25

**Authors:** Jamal Hussen, Omar A. AL-Jabr, Mayyadah Abdullah Alkuwayti, Noof Abdulrahman Alrabiah, Baraa Falemban, Abdulaziz Alouffi, Waleed S. Al Salim, Ketsarin Kamyingkird, Marc Desquesnes

**Affiliations:** 1 Department of Microbiology, College of Veterinary Medicine, King Faisal University, Al-Ahsa 31982, Saudi Arabia; oaljabr@kfu.edu.sa (O.A.A.-J.); bfalemban@kfu.edu.sa (B.F.); 2 Central Veterinary Laboratory, Ministry of Environment, Water and Agriculture, Riyadh 11195, Saudi Arabia; asn1950r@gmail.com (A.A.); waleed-alsalem@hotmail.com (W.S.A.S.); 3 Department of Biological Sciences, College of Science, King Faisal University, Al-Ahsa 31982, Saudi Arabia; malkwaiti@kfu.edu.sa (M.A.A.); nalrbeah@kfu.edu.sa (N.A.A.); 4 King Abdulaziz City for Science and Technology, Riyadh 12354, Saudi Arabia; 5 Ministry of Environment, Water and Agriculture, Riyadh 11195, Saudi Arabia; 6 Department of Parasitology, Faculty of Veterinary Medicine, Kasetsart University, Ladyao, Chatuchak, Bangkok 10900, Thailand; ketsarin.ka@ku.th; 7 CIRAD, UMR INTERTRYP, Ecole Nationale Vétérinaire de Toulouse (ENVT), 31300 Toulouse, France; marc.desquesnes@cirad.fr; 8 Interactions Hosts-Vectors-Parasites-Environment in the Tropical Neglected Disease due to Trypanosoma-Tids (INTERTRYP), University Montpellier, 34398 Montpellier, France

**Keywords:** *Trypanosoma evansi*, camel, phagocytosis, neutrophils, NETosis, flow cytometry

## Abstract

Surra, a wasting disease caused by *Trypanosoma evansi*, is one of the major animal health burdens in camel-rearing countries, imposing significant economic losses due to reduced fertility and high mortality rates. The present study used inactivated *T. evansi* (from the Card Agglutination Test for Trypanosomes/*Trypanosoma evansi*; CATT/*T. evansi*) and flow cytometry to investigate their binding and activation potential toward camel leukocyte subsets. Labeling *T. evansi* with propidium iodide (PI) enabled their flow cytometric enumeration and identification with forward scatter (FSC; indicative for cell size) and side scatter (SSC; indicative for cell internal complexity) characteristics that are comparable with values reported for *Trypanosoma cruzi*. The incubation of PI-labeled non-opsonized *T. evansi* with camel leukocyte populations revealed that camel monocytes have the highest potential to bind *T. evansi*, followed by granulocytes and lymphocytes. The identification of pattern recognition receptors (PRRs) on camel immune cells and the pathogen-associated molecular patterns (PAMPs) in *T. evansi* that are responsible for this different binding capacity requires further studies. Stimulation of camel neutrophils with *Trypanosoma evansi* induced shape change, reactive oxygen species (ROS) production, and neutrophil extracellular traps (NET)-formation. To ensure that *T. evansi*, in the parasite concentration used in this study, is not apoptotic or necrotic to camel leukocytes, we evaluated cell apoptosis and necrosis after stimulation with *T. evansi*. The results revealed no impact of *T. evansi* stimulation for 2 h on the cell viability of camel leukocytes. Subsequent work may focus on the diagnostic employment of labeled *T. evansi* and flow cytometry for the detection of anti-*Trypanosoma* antibodies in camel serum. In addition, more efforts should be deployed to investigate the host–pathogen interaction mechanisms and the escape mechanisms of *T. evansi* in camels. To complete these data, further studies using the living or freshly killed parasites could also be implemented in camels and/or horses.

## 1. Introduction

*Trypanosoma evansi* is responsible for an arthropod-borne disease known as Surra [1]. It is a blood parasite that is genetically partially derived from *Trypanosoma brucei* [2,3]. The disease caused by *T. evansi* affects several animal species with an especially high impact on camels (dromedary and Bactrian) [4,5,6] and equids [7], imposing significant economic losses due to reduced fertility and high mortality rates [3,8]. Besides *T. brucei* and *T. equiperdum*, *T. evansi* belongs to the subgenus *Trypanozoon* [9]. Several recent phylogenetic and immunologic studies reported high genetic and antigenic similarities between *T. evansi*, *T. equiperdum*, and *T. brucei* [10,11,12], suggesting a close relationship between the three parasites species and leading to the assumption that *T. evansi* and *T. equiperdum* could be considered a subspecies of *T. brucei* [9,13].

The world population of one-humped camel (*Camelus dromedarius*) has been estimated to reach more than 38.6 million head [14]. Under the current situation of increased global warming and the capacity of camels to survive under harsh climatic conditions and still produce milk and meat for human consumption, there has been increased interest in the improvement in camel health and production [3,15]. In recent years, several phenotypic and functional properties of camel immune cells were characterized in healthy and diseased animals [16]. In addition, the impact of several microbial Toll-like receptor (TLR)-agonists on selected phenotypic and functional properties of camel immune cells has been investigated [17].

Innate immune cells like monocytes, macrophages, and neutrophils are cells of the first line of defense with major roles in the sensing and early elimination of pathogens [18,19]. Their response to trypanosome infection is initiated by the recognition of pathogen structures, known as pathogen-associated molecular patterns (PAMPs), like the variable surface glycoprotein (VSG), using their pattern recognition receptors (PRRs) like TLR [20,21,22].

The lack of effective vaccines against trypanosomal infections [23], together with the increased development of drug-resistant strains, strengthens the need for a better understanding of the pathological and immunological mechanisms involved in *Trypanosoma* infections, paving the way for the development of prophylactic vaccines. Studies on the immune response of camels to *T. evansi* are still limited to serological detection of specific antibodies in the serums of infected animals [24,25], while the role of the cellular immune response has not been investigated so far. The aim of the present study was to investigate the impact of in vitro stimulation with inactivated *T. evansi* on selected functions of camel leukocytes.

## 2. Materials and Methods

### 2.1. Animal Use and Blood Sample Collection

Blood samples were collected from apparently healthy dromedary camels (*Camelus dromedarius*) aged between 9 and 11 years. The animals were reared at the farm of the Camel Research Center of King Faisal University, Al-Ahsa, Saudi Arabia. Blood samples were obtained by venipuncture of the jugular vein (vena jugularis externa) into vacutainer tubes containing EDTA (Becton Dickinson, Heidelberg, Germany). Animals ethical approval was obtained from the Ethics Committee of King Faisal University, Saudi Arabia, with an approval number (KFU-REC-2021- DEC -EA000326).

### 2.2. Separation of Mononuclear Cells from Camel Blood

Camel peripheral blood mononuclear cells (PBMCs) were separated from buffy-coat blood by density gradient centrifugation over Lymphoprep™ (STEMCELL Technologies, Vancouver, BC, Canada). For this, 5 mL of blood was diluted 1:2 with phosphate-buffered saline (PBS), and the mixture was layered on 5 mL of Lymphoprep™ in a 15 mL sterile falcon tube. The PBMC-containing interphase was collected carefully using a 10 mL pipette after centrifugation at 800× *g* for 30 min at 4 °C. The PBMCs were washed with cold PBS by centrifugation three times at 400× *g*, 200× *g*, and 100× *g*, respectively, for 10 min at 4 °C. The PBMC cells were counted and suspended in RPMI culture medium at 2 × 10^6^ cells/mL for evaluation of the cell purity and vitality (Supplementary Figure S1), as previously described [26].

### 2.3. Separation of Granulocytes from Camel Blood

After collection of the interphase containing the PBMC, camel granulocytes were separated from the remaining pellet via red blood cell (RBC) hypotonic lysis method. Ten milliliters of cold distilled water were added to the RBC-containing pellet for 20 s, and then 10 mL 2X-concentrated PBS was added and centrifuged at 500× *g* for 10 min at 4 °C. The lysis procedure was repeated until complete removal of the RBCs. Finally, granulocytes were counted and suspended in an RPMI culture medium at 2 × 10^6^ cells/mL for evaluation of the cell purity and vitality (Supplementary Figure S1).

### 2.4. Labeling of T. evansi with Propidium Iodide

A flask of freeze-dried purified (on DE52), formalin-fixed, and stained trypanosomes (using coomassie brilliant blue) of the Variable Antigen Type (VAT) Rode Trypanozoon antigen type (RoTat) 1.2 from the kit of Card Agglutination Test for *Trypanosoma evansi* (CATT/*T. evansi*) (OIE-Reference Laboratory for Surra, Institute of Tropical Medicine, Antwerp, Belgium) was reconstituted with 1 mL PBS, and the suspension was incubated with propidium iodide (PI; 2 μg/mL, Calbiochem, Darmstadt, Germany) for five minutes at RT and washed with cold PBS for 10 min. The labeled parasites were suspended in RPMI medium, counted on the flow cytometer (Accuri C6; Becton, Dickinson and Company, San Diego, CA, USA), and adjusted to a density of 4 × 10^6^ parasite/mL [27,28].

### 2.5. In Vitro Stimulation of Camel Neutrophils and PBMCs with T. evansi

Purified camel neutrophils or PBMCs were incubated in vitro with PI-labeled or unlabeled *T. evansi* for 30 min at 37 °C and 5% CO_2_. Briefly, 50 microliters containing *T. evansi* of 1 × 10^5^ trypomastigotes and 50 microliters of 1 × 10^5^ PBMCs cells in RPMI medium were added and incubated in a 96-well sterile cell culture plate. Incubation of PBMC cells without *T. evansi* in the medium was used as a control. In some experiments, *Staphylococcus aureus* (*S. aureus*) bacteria were used for control stimulation.

### 2.6. Analysis of Stimulation-induced Shape Change in Neutrophils

The change in cell size was determined by flow cytometric measurement of changes in the forward light scatter (FSC) axis of the neutrophils (Accuri C6 flow cytometer; Becton, Dickinson and Company, San Diego, CA, USA). Mean FSC values for stimulated neutrophils were compared with mean FSC values of unstimulated cells in the medium control. Overlapping histograms were generated using the C flow software (Version 1.0.264.21, Becton, Dickinson and Company, San Diego, CA, USA) to make a graphical comparison.

### 2.7. Generation of Reactive Oxygen Species (ROS)

The production of ROS metabolites was measured in 96-well round-bottom microtiter plates (Corning, NY, USA) as previously described [29]. Camel granulocytes (1 × 10^6^/100 μL/well) were incubated in 50 μL RPMI culture medium alone or in a medium containing *T. evansi* (4 × 10^6^ parasite/mL) for 30 min (37 °C, 5% CO_2_). After 15 min of incubation, dihydrorhodamine (DHR) 123 (Mobitec, Goettingen, Germany) was added to the cells at a final concentration of 750 ng/mL. The cells were washed in RPMI medium, and ROS production was analyzed by flow cytometry.

### 2.8. Cell Vitality Assay

Cell apoptosis and necrosis were analyzed using the Annexin V-FITC Apoptosis Staining/Detection Kit following the manufacturer’s protocol (Abcam, Cambridge, MA, USA; ab14085). Culture 96-well plates containing unstimulated and *T. evansi*-stimulated neutrophils or PBMCs (1 × 10^6^ cells in 100 μL RPMI cell culture medium) were centrifuged (1200 rpm for 3 min at RT), and 100 μL of KIT buffer containing 1:100 Annexin V-FITC and 1:100 PI were added to the cell pellet after removal of the supernatant. After incubation for five min at RT in the dark, the cells were acquired on the flow cytometer (BD Accuri C6 flow cytometer). Apoptotic cells (Annexin V positive/PI negative) were differentiated from necrotic (Annexin V positive/PI positive) and viable cells (Annexin V negative/PI negative) based on their emission in the green (FL1) and orange fluorescence channels (FL2) upon excitation at 488 nm [29].

### 2.9. Statistical Analyses

Statistical analysis was performed using Prism version 5 (GraphPad Software, San Diego, CA, USA). Data normality was evaluated using the Shapiro–Wilk method. Results were presented as mean ± standard error of the mean (SEM). Differences between the means of the two groups were tested with Student’s *t*-test or with one-factorial analysis of variance (ANOVA) and Bonferroni’s correction for more than two groups. Results were considered significant at a *p*-value of less than 0.05. The Kruskal–Wallis test was used as a non-parametric test in combination with Dunn’s Multiple Comparison test for comparison between means where the values were not normally distributed.

## 3. Results

### 3.1. Flow Cytometric Light Scatter Properties of T. evansi

**Figure 1:**
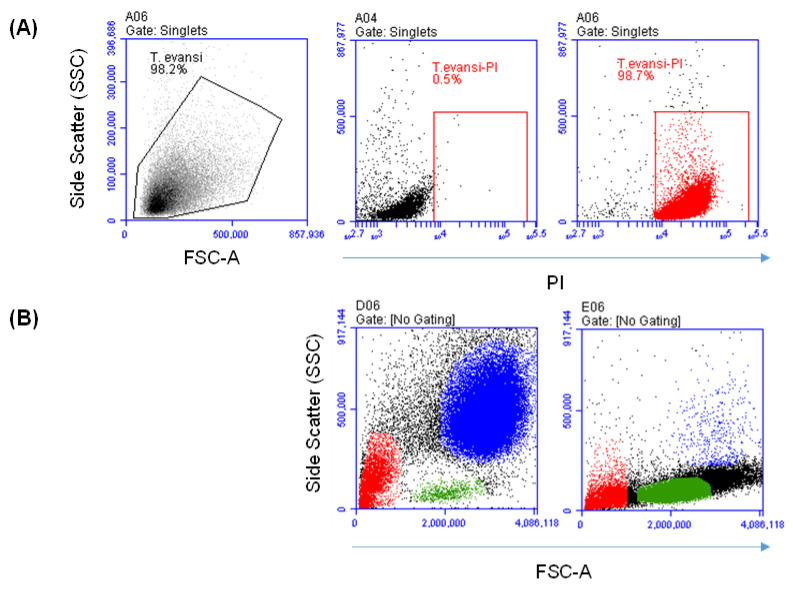

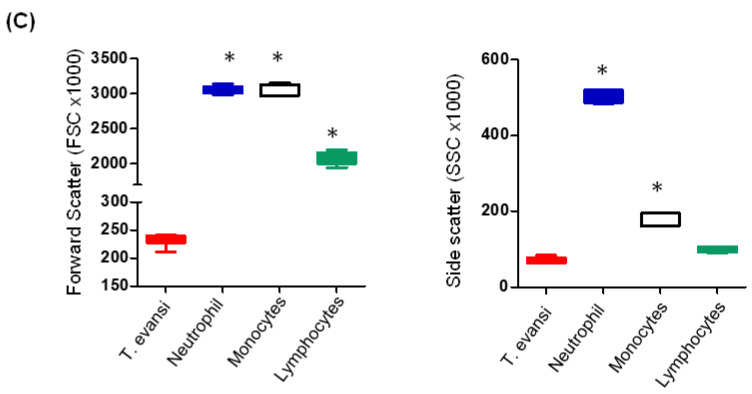
Flow cytometric identification of *Trypanosoma evansi* (*T. evansi*). Purified and formalin-fixed *T. evansi* were labeled with propidium iodide (PI), and labeled parasites were analyzed via flow cytometry. (**A**) Representative dot plot of FSC-A against SSC-A of *T. evansi*. After setting a gate on events in the FSC/SSC dot plot, PI-positive events were shown in a separate SSC-A against PI for labeled and unlabeled *T. evansi*. (**B**) Separated camel granulocytes or mononuclear cells were mixed with PI-labeled *T. evansi*. Colored FSC-A against SSC-A dot plots show the position of camel neutrophils (blue), monocytes (black), lymphocytes (green), and *T. evansi* (red). (**C**) FSC and SSC values were calculated and presented for *T. evansi* and camel leukocyte populations (n = 6 animals; * indicates *p* values less than 0.05 according to one-way ANOVA).

For flow cytometric identification of *T. evansi*, freeze-dried, purified (on DE52), formalin-fixed, and stained trypanosomes (using coomassie brilliant blue) of the Variable Antigen Type (VAT) Rode Trypanozoon antigen type (RoTat) 1.2 from the kit of Card Agglutination Test for *Trypanosoma evansi* (CATT/*T. evansi*) (OIE-Reference Laboratory for Surra, Institute of Tropical Medicine, Antwerp, Belgium) were labeled with propidium iodide (PI). As a DNA-binding dye that can only penetrate the membrane of dead cells, labeling with PI enabled the identification of *T. evansi* as PI-positive events (Figure 1A). After their identification, the FSC and SSC mean values were calculated and compared with the mean values of leukocyte populations (Figure 1B). With an FSC value of 230.6 ± 3.7 × 10^3^ (Mean ± SEM), *T. evansi* showed a significantly (*p* < 0.05) lower cell size than all blood leukocyte populations. The cell granularity indicator (SSC) value of 69.4 ± 3.8 × 10^3^ of *T. evansi* was only lower than that of neutrophils and monocytes but comparable (*p* > 0.05) with lymphocytes SSC (Figure 1C).

### 3.2. Binding Potential of T. evansi to Camel Granulocytes and Mononuclear Cells

**Figure 2:**
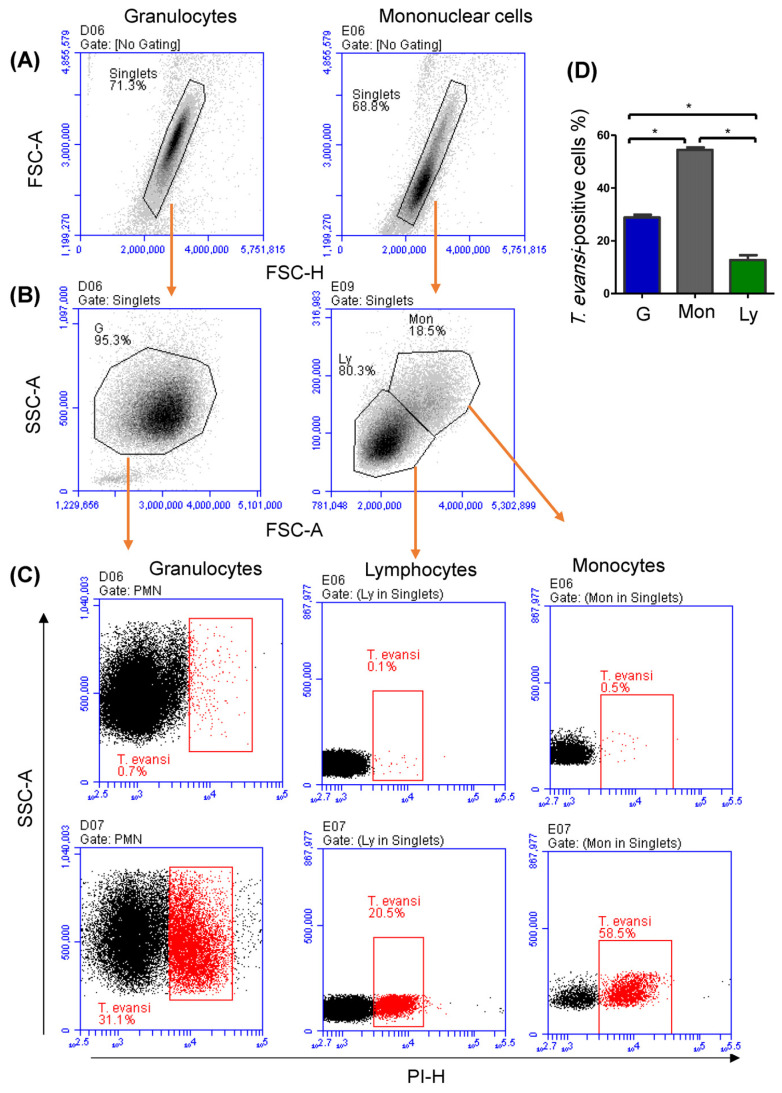
Binding potential of *T. evansi* to camel leukocyte populations. Granulocytes and mononuclear cells (PBMC) were separated from camel peripheral blood and were incubated in vitro with PI-labeled *T. evansi*. (**A**) After exclusion of cell duplets in a FSC height (FSC-H) against FSC area (FSC-A) dot plot, (**B**) granulocytes (G), monocytes (Mon), and lymphocytes (Ly) were gated based on their FSC and SSC characteristic. (**C**) The percentage of cells with increased staining with PI was calculated for cells incubated in medium alone or in medium containing *T. evansi*. Mean and SEM values were presented (**D**) graphically (n = 11 animals; * indicates significant differences based on a *p* value less than 0.05 according to one-way ANOVA).

The binding potential of *T. evansi* to the different populations of camel leukocytes was analyzed by flow cytometry after labeling the dead trypanosomes with PI (Figure 2A). With a mean percentage of cells that bound to *T. evansi* after in vitro incubation of 54.5% ± 0.9 (SEM), camel monocytes showed the highest (*p* < 0.05) binding potential, followed by granulocytes (28.8% ± 1.0), while lymphocytes (12.7 ± 1.8%) showed the lowest (*p* < 0.05) percentage of *T. evansi*-binding cells (Figure 2B).

### 3.3. Stimulation Potential of T. evansi on Camel Neutrophils

**Figure 3:**
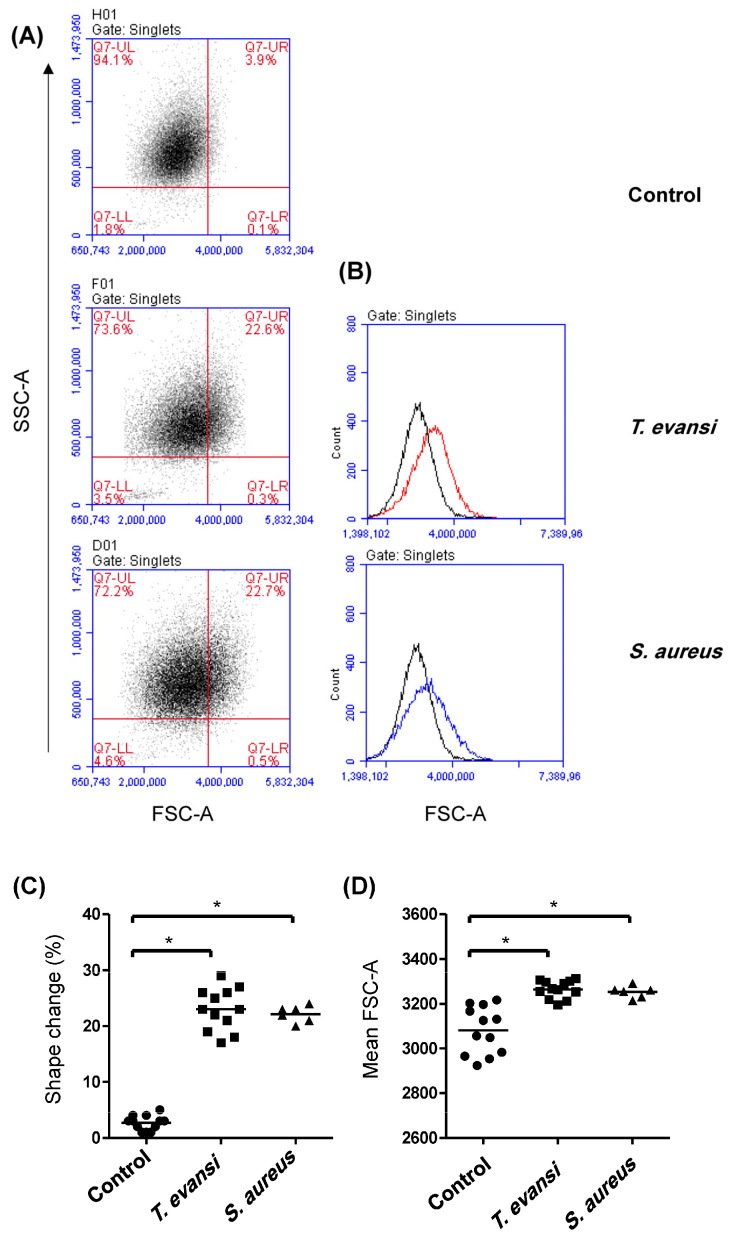
Stimulation-induced shape change in camel neutrophils. Separated camel neutrophils were incubated with *T. evansi* for 1 h and their shape change was analyzed by the measurement of change in their cell size (FSC-A). (**A**) After exclusion of cell duplets, the percentage of cells with increased FSC-A was calculated in the upper right quadrant of an FSC-A against an SSC-A dot plot. (**B**) In addition, the change in the FSC-A mean fluorescence intensity (MFI) of the whole cell population was presented in a histogram comparing *T. evansi*- (red line), *S.aureus*-stimulated (blue line) and unstimulated control cells (black line). The percentage of cells with shape change (**C**), as well as the change in FSC-A (**D**), were presented for cells incubated in medium alone or in medium containing *T. evansi* or *S. aureus*. * indicates significant differences between the groups.

The potential of *T. evansi* to stimulate camel neutrophils was evaluated based on the stimulation-induced shape change (cell size) in neutrophils upon in vitro incubation with *T. evansi* (Figure 3A). The effect of *T. evansi* was compared with the shape change induced after stimulation with *Staphylococcus aureus* bacteria. In comparison to neutrophils incubated in medium control alone (2.3% ± 0.3, stimulation with either *T. evansi* (23.0% ± 0.3) or *S. aureus* (22.1% ± 0.6) resulted in a significant (*p* < 0.05) increase in the fraction of cells with induced shape change (increased cell size as measured by the analysis of FSC) (Figure 3B,C). Similarly, the mean fluorescence intensity (MFI) of FSC-A was significantly higher (*p* < 0.05) for cells stimulated with *T. evansi* or *S. aureus* than control cells (Figure 3D).

### 3.4. In Vitro Stimulation with T. evansi-Induced ROS Production in Camel Neutrophils

**Figure 4:**
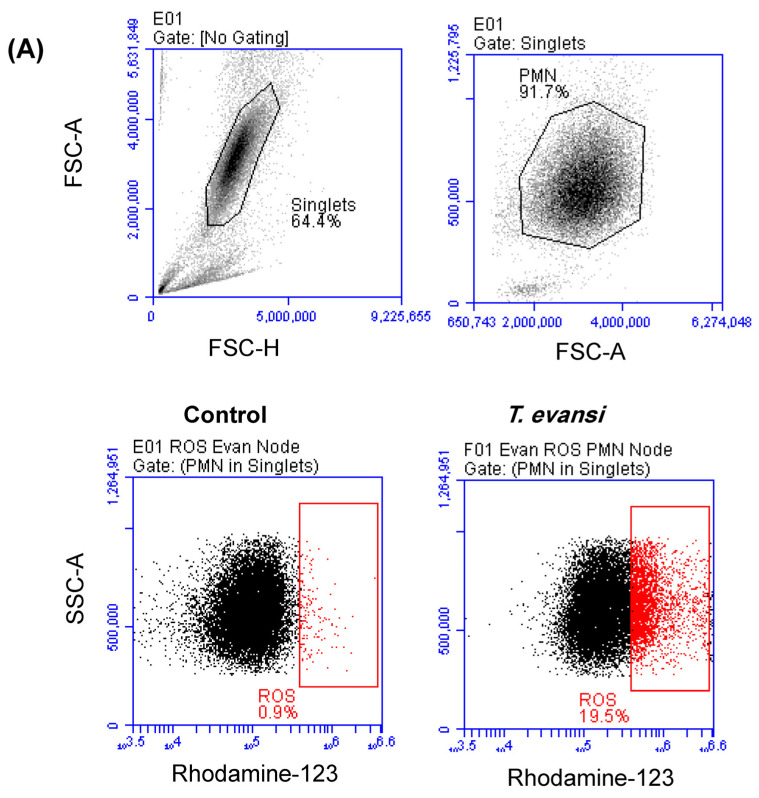

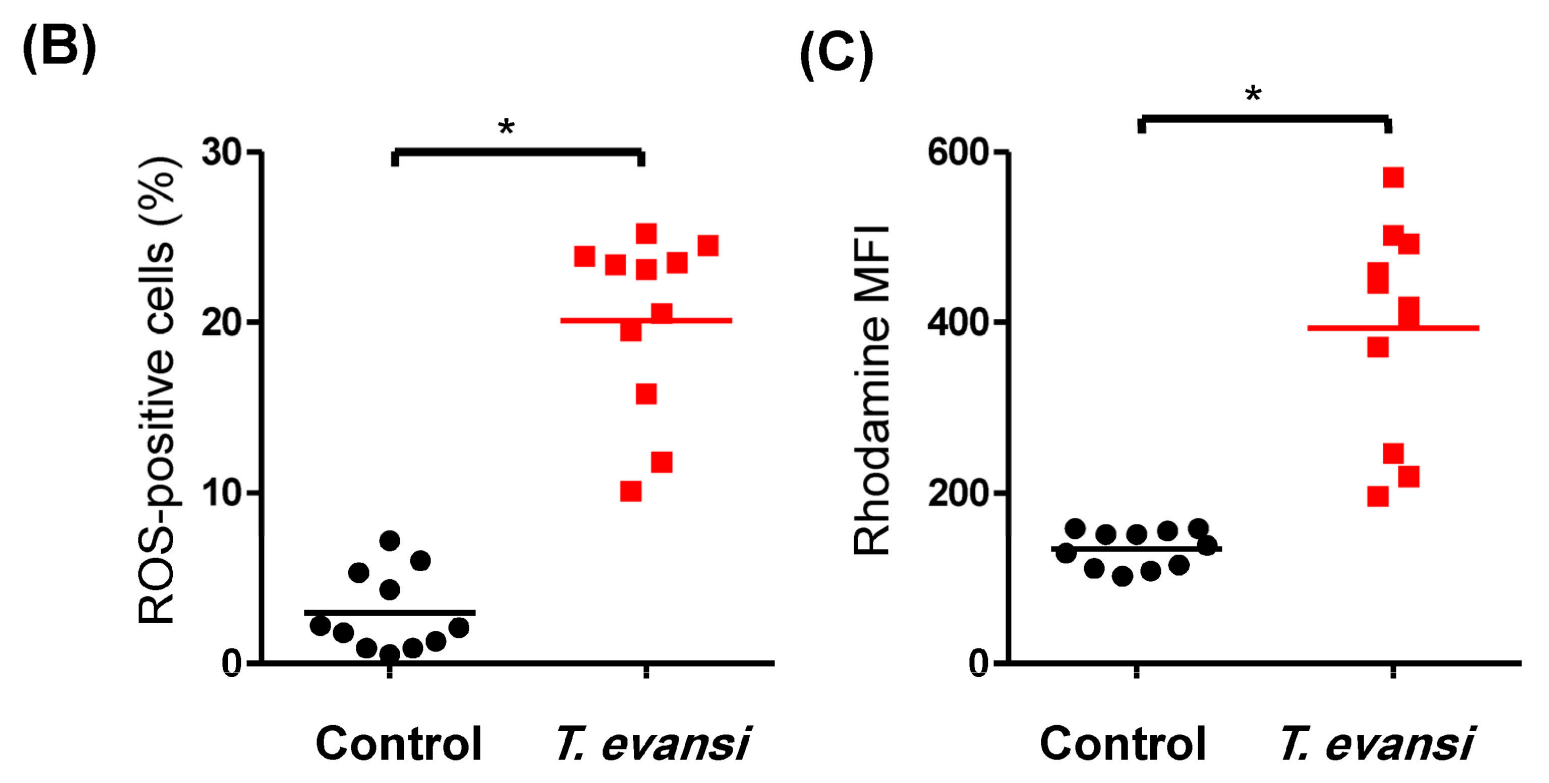
ROS production in camel neutrophils after in vitro stimulation with *T. evansi*. Separated camel neutrophils were incubated in medium control alone or in the presence of *T. evansi* parasites. The ROS-sensitive dye Dehydrorohdamine-123 (DHR-123) was added to the cells 15 min after incubation. (**A**) After setting gates on single cells and granulocytes (black gates), the percentage of cells with increased Rhodamine fluorescence (red gates) was calculated within the neutrophils population (PMN). (**B**) the percentage of cells with ROS production, as well as the MFI of Rhodamine (**C**), were presented for stimulated and unstimulated cells (n = 11 animals). * indicates significant differences with *p* value less than 0.5 according to Student’s *t*-test.

The potential of *T. evansi* to induce the production of ROS in camel neutrophils was analyzed by flow cytometry (Figure 4A). In comparison to cells incubated in the medium alone, incubation with *T. evansi* resulted in a significant (*p* < 0.05) increase in the percentage of neutrophils that produced ROS (20.1 ± 1.5% versus 2.9 ± 0.7% for unstimulated cells) (Figure 4B). Similarly, the MFI of the ROS-sensitive dye DHR was three times higher for cells stimulated with *T. evansi* (393.1 ± 37 × 10^3^) than unstimulated cells (134.3 ± 6.7 × 10^3^) (Figure 4C).

### 3.5. In Vitro Stimulation with T. evansi-Induced Neutrophil Extracellular Traps (NET)-Formation in Camel Neutrophils

**Figure 5:**
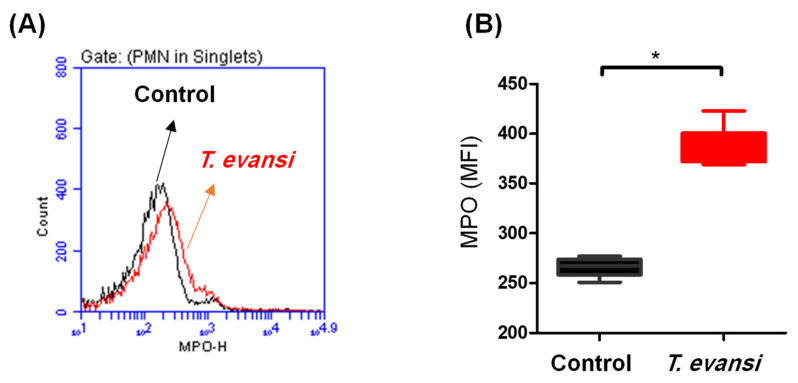
NET-formation in camel neutrophils after in vitro stimulation with *T. evansi*. Separated camel neutrophils were incubated in medium control alone or in the presence of *T. evansi* parasites. After incubation, cells were stained with monoclonal antibodies to myeloperoxidase (MPO) and analyzed by flow cytometry. (**A**) Representative histogram showing staining of stimulated and unstimulated cells with anti-MPO antibodies. (**B**) The MFI of MPO was calculated and presented for stimulated and unstimulated cells (n = 6 animals). * indicates significant differences with *p* value less than 0.5 according to Student’s *t*-test.

NET-formation was analyzed by flow cytometric detection of the neutrophil’s granular enzyme myeloperoxidase (Figure 5A). Stimulation of camel neutrophils with *T. evansi* induced a significant (*p* < 0.05) production of MPO (385.5 ± 8.2 MFI of anti-MPO antibody) in comparison to unstimulated control cells (266.2 ± 3.8 MFI of anti-MPO antibody) (Figure 5B).

### 3.6. Stimulation with T. evansi Did Not Impact the Vitality of Camel Neutrophils

**Figure 6:**
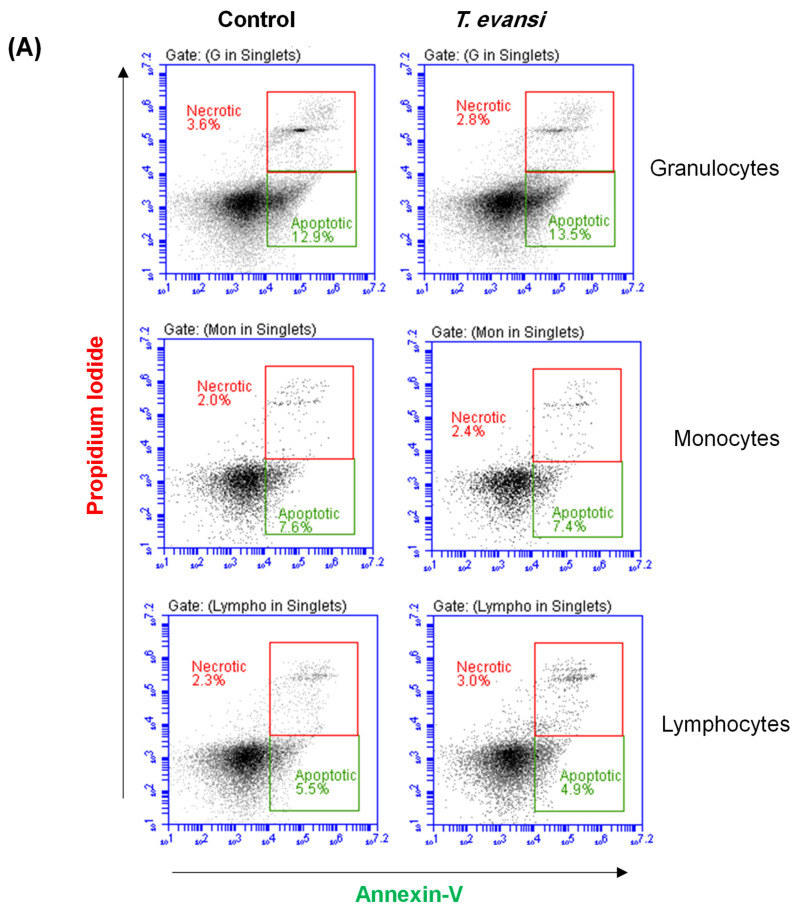

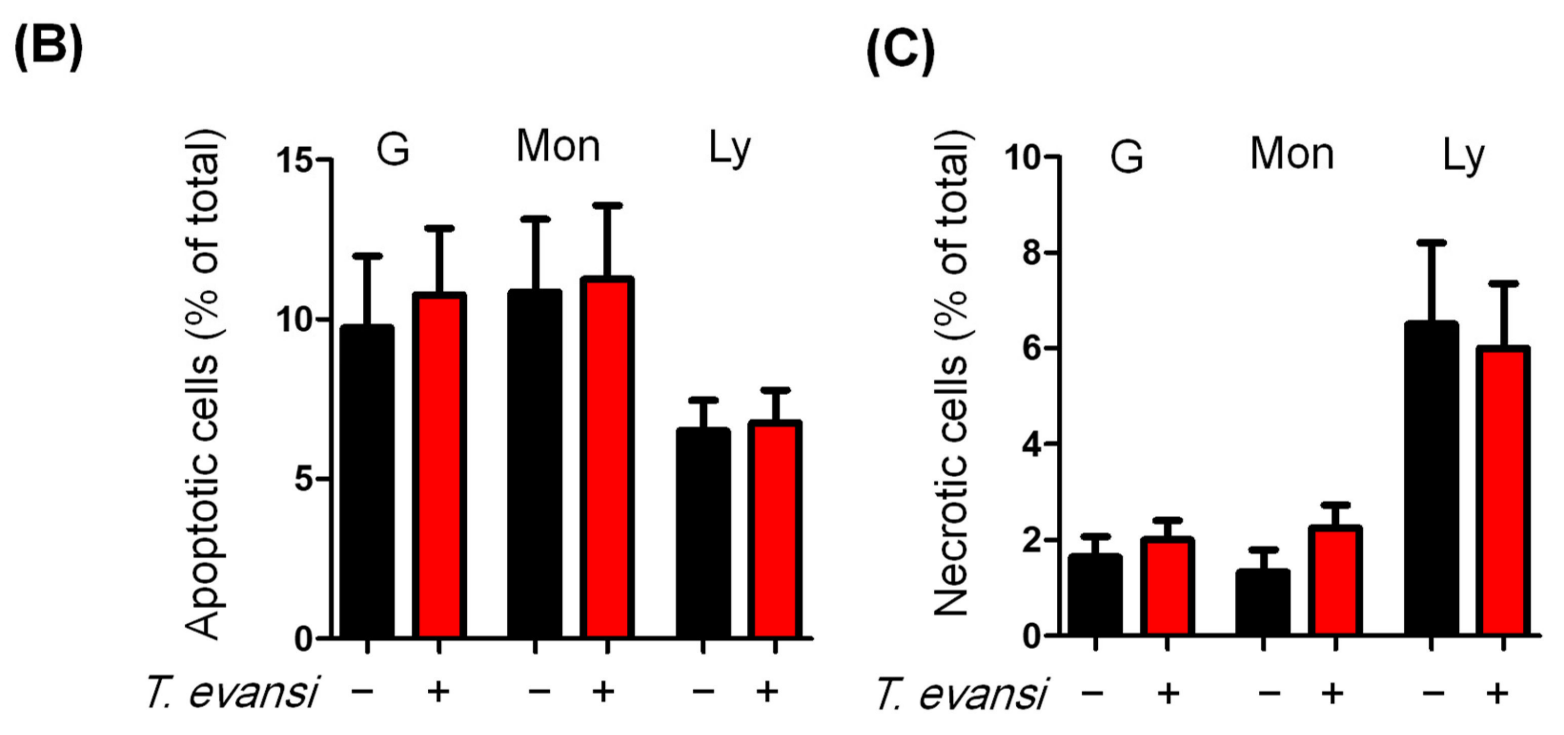
Analysis of cell apoptosis and necrosis. Camel granulocytes or mononuclear cells were incubated in medium control with or without *T. evansi* for 2 h in vitro, followed by staining with a combination of Annexin V and PI for the detection of cell apoptosis and necrosis, respectively. (**A**) Annexin V against PI dot plots for the identification of apoptotic (Annexin+/PI^−^ and necrotic (Annexin-/PI+) cells. The percentage of apoptotic (**B**) and necrotic (**C**) cells within camel granulocytes, monocytes, or lymphocytes were presented for stimulated and unstimulated cells (n = 4 animals).

The impact of *T. evansi* stimulation on camel granulocytes, monocytes, and lymphocytes cell vitality was evaluated by flow cytometric measurement of cell apoptosis and necrosis (Figure 6A). For cells incubated for 2 h in medium control alone, spontaneous apoptosis resulted in a percentage of 9.7 ± 2.2%, 10.8 ± 2.1%, and 6.5 ± 0.9% of total granulocytes, monocytes, and lymphocytes, respectively (Figure 6B). The percentage of necrotic cells was 1.6 ± 0.4%, 1.3 ± 0.4%, and 6.5 ± 1.6% of total granulocytes, monocytes, and lymphocytes, respectively (Figure 6C). Stimulation with *T. evansi* for 2 h did not induce any significant changes in the percentage of apoptotic or necrotic cells within camel granulocytes, monocytes, or lymphocytes (Figure 6A–C).

## 4. Discussion

Surra is a disease caused by the blood parasite *T. evansi* that affects various animal species. Although it is responsible for high mortality and economic losses and is a World Animal Health Organization (WOAH) listed disease, surra is still a neglected disease in terms of research into improved prevention and control strategies [30,31]. Especially in camel *T. evansi* infection, studies on the innate and adaptive immune responses, the host–pathogen interaction mechanisms, and immune evasion strategies of the parasite are very limited [2,3].

In the present study, we labeled inactivated *T. evansi* with the DNA-sensitive dye PI, which penetrates the membrane of dead cells, resulting in the enhancement of its fluorescence signal 20 to 30 folds upon DNA binding [32]. Labeling with PI enabled the identification of FSC and SSC characteristics of *T. evansi*, as well as flow cytometric enumeration of the parasites before using it for in vitro activation studies. The FSC and SSC values of *T. evansi*, which allow the discrimination of cells based on cell size and internal complexity or granularity, respectively [33], seem to be comparable with light scattering values reported in previous studies for *T. cruzi* [34]. The employment of flow cytometry in the diagnosis of trypanosomosis via the immunodetection of *Trypanosoma*-specific antibodies in human and canine serum has been described previously [35,36]. Subsequent work could focus on using labeled *T. evansi* and flow cytometry for the detection of anti-*Trypanosoma* antibodies in camel or horse serum using fluorochrome-labeled anti-species IgG antibodies. In this approach, *T. evansi* will be incubated with serum from infected camels or horses, followed by the addition of anti-camel or anti-horse IgG labeled with fluorochrome to detect the antibodies bound on the parasite. Although the limited availability of flow cytometers at every laboratory and the high cost of using fluorochrome-labeled antibodies represent challenges for the diagnostic application of this technique, such an approach is expected to provide higher-sensitive detection of the serologic response against *T. evansi*, which could be used for confirmatory diagnosis of doubtful cases. In addition, the value of flow cytometry as a method for studying host–parasite interaction mechanisms has been demonstrated in several trypanosomal studies. For instance, Schulz et al. used flow cytometry for the analysis of trypanosome VSG antigen switching rate in a given population by the identification and enumeration of parasites that express a new VSG [37]. Similarly, Miranda et al. established a flow cytometry method to study the interactions between *Trypanosoma* parasites, the anti-trypanosomiasis drug, and host cells [38]. Therefore, flow cytometry represents a potential tool for trypanosome research in camels and horses that could be useful to measure or estimate the capacity of a host to identify and therefore control the parasite (host resistance or trypanotolerance).

To ensure that *T. evansi*, at the concentration used in this study, is not apoptotic or necrotic to camel leukocytes, we evaluated cell apoptosis and necrosis after stimulation with *T. evansi*. The results revealed no impact of *T. evansi* stimulation for 2 h on the cell viability of camel leukocytes.

**Figure 7:**
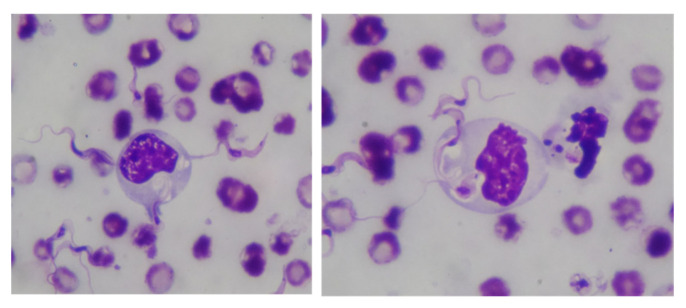
Illustration of the binding and phagocytosis activity of monocytes toward *T. evansi* in horse blood. Blood smears were prepared from horse blood, stained with Giemsa stain, and analyzed under a microscope (immersion oil 1000×).

The early immune response to protozoan parasite infections is triggered after the recognition of protozoan PAMPs by innate PRRs, leading to the activation of several cell types of the innate immune system, such as neutrophils and macrophages and the subsequent initiation of adaptive immune mechanisms. In recent studies, several protozoan PAMPs that activate the host TLRs were described, arguing for the vital role of TLR signaling pathways in the development of protective or pathologic immune responses to protozoan infections [39]. In the present study, the incubation of PI-labeled non-opsonized *T. evansi* with camel leukocyte populations revealed that camel monocytes have the highest potential to bind *T. evansi*, followed by granulocytes and lymphocytes. In a recent work, Kamyingkird et al., reported a special role for blood monocytes in the interaction with *T. evansi*. The study identified a vesicle-like form of *T. evansi* in monocytes from horses with a Surra outbreak [40]. This indicates the potential of monocytes to bind and interact with *T. evansi*, as shown in Figure 7 prepared from the same study (unpublished data with permission from Dr. Kamyingkird and Dr. Marc Desquesnes). The identification of PRR on camel immune cells and the PAMPs on *T. evansi* that are responsible for this different binding capacity requires further studies. Studies on other *Trypanosoma* species identified some key TLR and TLR-ligands that are involved in the innate sensing of *Trypanosoma*. This includes the role of TLR2 in the recognition of alkyl-acyl-glycerol in *T. cruzi* and lipo-phospho-glycan in *Leishmania*, binding of glycosyl-phosphatidyl-inositol in *Trypanosoma* species by TLR2/TLR4, and sensing trypanosomal DNA by TLR9 [41].

Neutrophils are key innate immune cells rapidly recruited to sites of infection, where they kill invading micro-organisms by several mechanisms, including phagocytosis and production of reactive oxygen species (ROS), degranulation, and NETosis (formation of neutrophils extracellular traps; NETs) [42]. Compared to other protozoal pathogens, studies on the role of neutrophils in the immune response to *Trypanosoma* species are limited [43,44,45]. To see whether the binding of *T. evansi* to camel neutrophils is associated with cell activation, we evaluated the stimulation-induced shape change in neutrophils in in vitro incubation with *T. evansi*. The results showed a comparable stimulatory effect of *T. evansi* to that of the Gram-positive bacteria *Staphylococcus aureus* (*S. aureus*). This was also confirmed by the significant increase in ROS production and the enhanced expression of the NETosis marker myeloperoxidase [46] in camel neutrophils upon in vitro stimulation with *T. evansi*. Further investigation is needed to clarify whether the production of ROS or the formation of NETs by camel neutrophils are involved in the disease pathogenesis or protective innate immune response to *T. evansi* in camels.

## 5. Conclusions

In summary, the present study used inactivated *T. evansi* to study its binding and activation potential toward camel leukocyte subsets. Labeling with PI enabled the flow cytometric enumeration and identification of *T. evansi* with FSC and SSC characteristics that are comparable with values reported for *T. cruzi.* Camel monocytes showed the highest potential to bind *T. evansi*, followed by granulocytes and lymphocytes. The identification of PRR on camel immune cells and the PAMPs on *T. evansi* that are responsible for this different binding capacity requires further studies. *Trypanosoma evansi* induced shape-change, ROS production, and NET-formation in camel neutrophils. Subsequent work may focus on the employment of labeled *T. evansi* and flow cytometry for host–pathogen interaction studies in camels or horses.

## Data Availability

The datasets generated during the current study are available from the corresponding author upon reasonable request.

## Supplementary Materials

The following supporting information can be downloaded at https://www.mdpi.com/article/10.3390/pathogens13010021/s1. Figure S1: Analysis of cell purity and vitality.
