# Supplementary material for: A Flow Cytometry Study of the Binding and Stimulation Potential of Inactivated Trypanosoma evansi toward Dromedary Camel Leukocytes
Source: Pathogens. 2023 Dec 25;13(1):21. doi: 10.3390/pathogens13010021 (PMC10820945; doi:10.3390/pathogens13010021)
Supplement: Supplementary file 1 [file pathogens-13-00021-s001.zip › pathogens-2744055-supplementary.pdf]

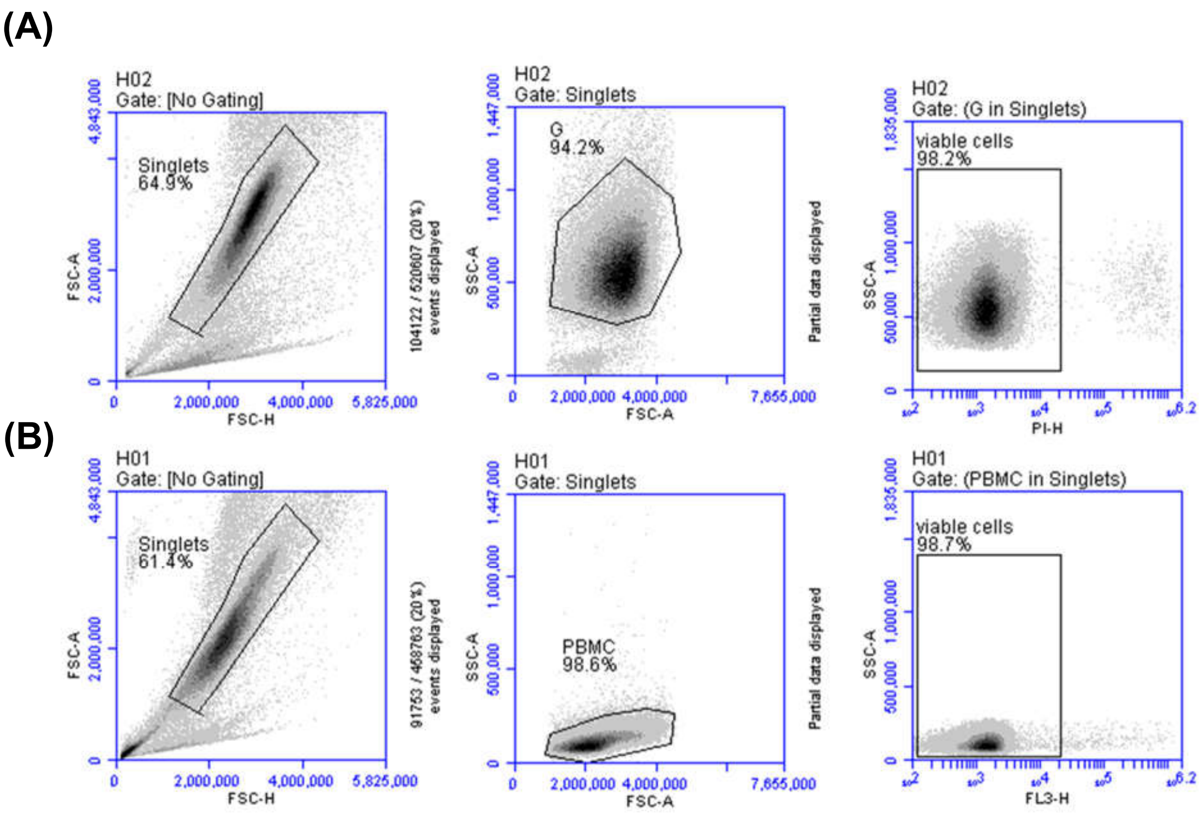

Supplementary S1: Evaluation of purity and vitality of separated cells. Camel blood granulocytes (**A**) or mononuclear cells (**B**) were labeled with propidium iodide (PI) and analyzed by flow cytometry. After gating on singlets, cell purity was measured based on FSC and SSC properties of granulocytes (G) and mononuclear cells (PBMC). Cell vitality was measured based on the negative staining with PI.
